# Quadratus Lumborum Block: The New Gold Standard in Abdominal Analgesia?

**DOI:** 10.7759/cureus.88051

**Published:** 2025-07-16

**Authors:** Hubert Rytel, Bestun Rashid, Peter Kaczmarski, Michael Kaczmarski, Ithamar Cheyne, Malgorzata Mikaszewska-Sokolewicz

**Affiliations:** 1 Department of Anesthesiology and Intensive Care, Children's Memorial Health Institute, Anaesthesiology and Intensive Care Scientific Circle English Division (ANKONA ED) Medical University of Warsaw, Warsaw, POL

**Keywords:** eras, ql block, quadratus lumborum, regional anesthesia, ultrasound

## Abstract

The quadratus lumborum block (QLB) is a common regional anesthesia technique for abdominal and pelvic surgeries. Unlike traditional blocks (e.g., transversus abdominis plane (TAP) block) that target only somatic pain, QLB provides both somatic and visceral analgesia by facilitating the spread of local anesthetic through the thoracolumbar fascia. Its use in enhanced recovery after surgery (ERAS) protocols has increased due to its effectiveness in reducing postoperative opioid consumption, enabling early mobilization, and minimizing systemic side effects. Compared to epidural and TAP blocks, QLB is less invasive and offers longer-lasting pain relief. However, its clinical use is limited due to the need for specialized training in ultrasound-guided techniques.

Recent studies have emphasized QLB's advantages over TAP and epidural blocks for cesarean sections, colorectal surgeries, and hip replacements. Meta-analyses repeatedly indicate that QLB leads to decreased postoperative opioid consumption, lower pain levels, and shorter hospital stays. Among the different methods, QLB-2 and QLB-3 provide the broadest coverage. Pediatric research also reports positive results, especially in procedures like pyeloplasty and laparoscopic appendectomy. Improvements in ultrasound technology have enhanced the safety and effectiveness of QLB, although challenges remain due to varying anesthetic dispersal.

This review finds that QLB offers considerable benefits for abdominal analgesia, making it a novel and promising technique for standardizing abdominal wall analgesia and establishing a new gold standard in this area. When combined with proper ultrasound training and broader clinical application, QLB could enhance perioperative pain management in different surgical populations. Continued research will be vital for standardizing techniques and broadening their application in multimodal analgesia protocols.

## Introduction and background

The quadratus lumborum block (QLB) is a relatively new and emerging regional anesthesia technique for managing perioperative pain in abdominal and pelvic surgeries. First described by anesthesiologist Blanco in 2007 as a variant of the transversus abdominis plane block, this method has quickly evolved with more approaches and ultrasound guidance to minimize complications and optimize pain control [1]. However, an ongoing debate exists regarding the optimal technique for administration, given the discrepancies in the affected area and the differences in enhanced recovery after surgery (ERAS) procedures. This review aims to summarize current literature on the various approaches, anatomy, mechanism of action, clinical applications, safety, and established protocols related to the QLB while also addressing potential areas for future research.

## Review

Anatomy of the quadratus lumborum block

The thoracolumbar fascia (TLF) is a sheet of fused aponeuroses and fascial layers encasing the posterior abdominal wall musculature and facilitating the cranial and caudal spread of local anesthetics that affect both somatic and visceral structures during QLB.

**Figure 1:**
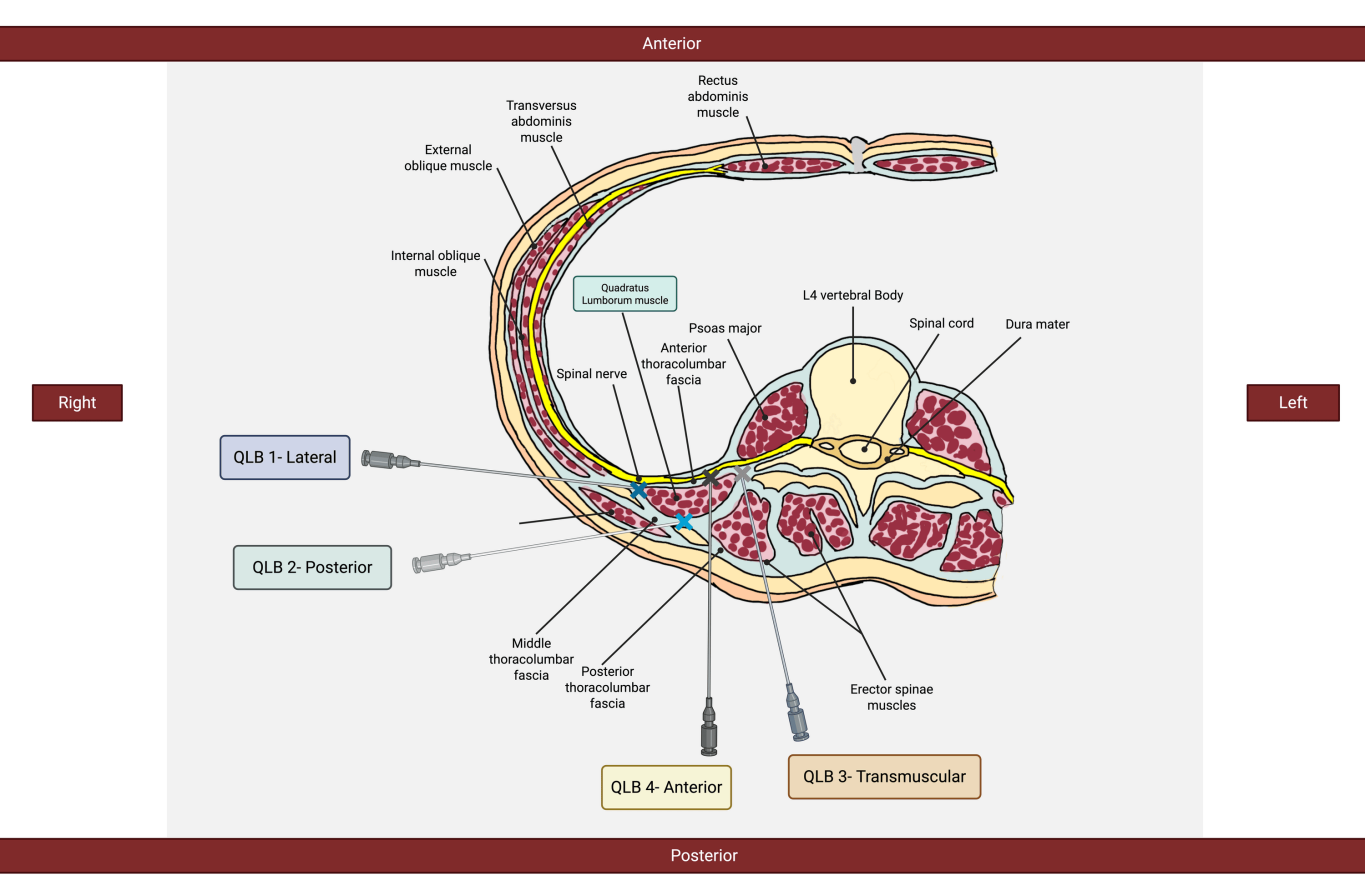
A cross-sectional anatomical illustration of the lumbar region, highlighting the four approaches to the quadratus lumborum block (QLB): QLB-1, QLB-2, QLB-3, and QLB-4. The techniques are shown in relation to key anatomical structures, including the quadratus lumborum muscle, thoracolumbar fascia, spinal nerves, and surrounding musculature. Created by the authors.

As illustrated in Figure 1, TLF comprises three distinct layers - anterior, middle, and posterior - organized around the musculature of the posterior abdominal wall [2]. The anterior layer lies anterior to the quadratus lumborum (QL) muscle, while the middle layer is situated between the QL and the erector spinae muscles. The posterior layer encloses the erector spinae muscles but not the QL. Medially, the anterior layer blends with the fascia of the psoas major (PM), and laterally it merges with the transversalis fascia [2]. When local anesthetic is injected between the anterior layer and the QL, it can spread cranially beneath the lateral arcuate ligament into the endothoracic fascia, potentially reaching the lower thoracic paravertebral space [2]. Recent anatomical studies have identified a triangular region - termed the lumbar interfascial triangle (LIFT) - located at the lateral border of the erector spinae, where the middle layer of the lumbar fascia merges with the deep lamina of the posterior layer (paraspinal retinacular sheath). This site is now considered an optimal target for injection to maximize anesthetic spread in QLB2 [3].

Due to the anatomical characteristics of the region, several methods of QLB exist: anterior, posterior, lateral, and transmuscular approaches. Different approaches result in variations in the spread of anesthetics. Therefore, each approach affects the block's duration and effectiveness [3]. Thus, the clinical context should be the deciding factor in choosing the approach.

Mechanism of action of the quadratus lumborum block

The primary reference point is TFL, which has anterior, middle, and posterior layers that facilitate the cranial and caudal distribution of the local anesthetic, along with blocks of somatic and visceral pain pathways. In somatic analgesia, QLB inhibits somatic pain by targeting the iliohypogastric, ilioinguinal, subcostal, lateral femoral cutaneous nerves, and the nerve roots T7 to L1 for somatic analgesia and T7 to L2 for sympathetic innervation, thereby providing effective pain relief in the abdominal wall [4]. In visceral analgesia, the local anesthetic, via the TLF, spreads to the thoracic paravertebral space, reaching the celiac plexus, splanchnic nerves, and sympathetic trunk [4].

The TFL fascia encompasses dense free nerve endings, including nociceptors, mechanoreceptors, and proprioceptors, which are involved in acute and chronic pain pathways. Injecting anesthetic agents into these high-density postganglionic sympathetic fibers allows for the modulation of high-threshold mechanoreceptors and nociceptors, reducing afferent pain signals [5]. Cranial anesthetic spread can reach the thoracic paravertebral space via the anterior layer of TFL up to T4-T5 dermatomes [3]. Caudally, the anesthetic spreads to the lumbar plexus and, in some approaches, to the sacral region [1].

Dosage of local anesthetics in quadratus lumborum block

The volume of local anesthetics used in QLB typically ranges from 20 to 30 mL per side, occasionally reaching up to 40 mL. A higher volume leads to greater cranial and caudal spread of the anesthetic, thereby increasing dermatome coverage [6,7]. Larger volumes permit a more extensive spread along the TLF, affecting more somatic and visceral nerve structures. However, an increased volume also raises the risk of local anesthetic systemic toxicity, which may result in cardiotoxicity and seizures [6,8].

The most commonly used anesthetic in QLB is ropivacaine (0.2%-0.375%) because it offers a longer duration and a lower risk of cardiotoxicity compared to another widely used anesthetic, bupivacaine (0.25%-0.5%) [6]. Additionally, lidocaine (0.5%-1%) is utilized for shorter, less invasive surgeries due to its rapid onset and shorter duration of action. Furthermore, some adjuvant drugs, such as epinephrine, are used to prolong effects, while others, like dexamethasone, are utilized to reduce inflammation [6,8].

Different approaches of the quadratus lumborum block

The primary variants of the QLB include QLB-1 (lateral), QLB-2 (posterior), QLB-3 (transmuscular), and QLB-4 (anterior/intramuscular). Each technique varies in injection site, patient positioning, ultrasound (US) view, and needle trajectory, influencing the spread of anesthetic and clinical application. Proper technique enhances block efficacy while minimizing complications like local anesthetic systemic toxicity (LAST) or inadvertent organ puncture. Figure 1 illustrates the anatomy and ultrasound-guided perspectives of QLB techniques, while Table 1 compares the QLB variants.

**Table 1:** Comparison of Quadratus Lumborum Block (QLB) Variants

QLB Type	Block Range	Spread Direction	Nerves Blocked	Common Clinical Applications	Analgesia Coverage	Advantages	Specific Risks	References
QLB-1 (lateral)	T7-L1	Lateral	Subcostal, iliohypogastric, ilioinguinal	Laparoscopic appendectomy, hernia repair, cholecystectomy	Somatic pain relief in the abdominal wall	Low complication rate, simple ultrasound guidance	Minimal; low risk with proper technique	[9-12]
QLB-2 (posterior)	T7-L1	Posterior, broad	Thoracolumbar dorsal rami	Cesarean section, bowel resection, gynecological surgeries	Somatic and visceral pain relief	Effective for both somatic and visceral pain, broad coverage	Vascular puncture, intraperitoneal injection	[1,8,10,13-15]
QLB-3 (transmuscular)	T7-L2	Cranial, caudal	Lumbar plexus	Colorectal surgeries, hip arthroplasty, pelvic procedures	Extensive somatic and visceral analgesia	Covers lower abdomen, pelvis, and lower extremities	Motor blockade, risk of lumbar plexus spread	[10, 11,13,16-19]
QLB-4 (anterior)	T7- T12	Anterior, broad	Thoracolumbar nerves	Laparoscopic and gynecological surgeries	Extensive somatic and visceral analgesia	Effective for laparoscopic and gynecological surgeries	Vascular injury, inadvertent intraperitoneal injection, deep anatomical approach	[1,11,15,17,20,21]

QLB-1 (lateral QLB) is typically performed with the patient in the supine position with a slight lateral tilt or in the lateral decubitus position [4]. A high-frequency linear or curved ultrasound probe is placed transversely at the mid-axillary line between the costal margin and iliac crest [9]. The external oblique, internal oblique, and transversus abdominis muscles are visualized, with the QL muscle deep to the transversalis fascia [4]. The injection targets the thoracolumbar fascia, allowing the anesthetic to spread and block the subcostal, iliohypogastric, and ilioinguinal nerves [10]. QLB-1 is primarily used in lateral abdominal wall surgeries such as laparoscopic appendectomy, hernia repair, and cholecystectomy. This approach effectively manages somatic pain due to its localized action on somatic nerves. QLB-1 has a low complication rate because of its relatively superficial injection site. However, ultrasound guidance is essential to minimize the risks of vascular puncture and intramuscular injection [10].

QLB-2 (posterior QLB) is usually done in the lateral decubitus position [11]. A low-frequency curvilinear probe is positioned more posteriorly than QLB-1 to visualize the posterior aspect of QL and the lumbar interfascial triangle, bordered by QL, latissimus dorsi, and erector spinae [11]. The needle is advanced in-plane from lateral to medial into the posterior thoracolumbar fascia, between QL and overlying paraspinal muscles [9]. This technique targets the dorsal rami and branches of the thoracolumbar nerves, enabling the anesthetic to spread along the posterior thoracolumbar fascia [12]. This approach provides broad somatic and visceral analgesia, making it suitable for cesarean sections, bowel resections, and gynecological surgeries because it can relieve pain in both somatic and visceral regions [1,13]. Because of its deeper anatomical target, QLB-2 has an increased risk of complications, including vascular puncture and inadvertent intraperitoneal injection [8].

QLB-3 (transmuscular QLB) is performed with the patient in the lateral position (side to be blocked facing up) [14]. The ultrasound probe is placed in a transverse orientation at the posterior axillary line [11]. The “shamrock sign” is identified: QL laterally, psoas major anteriorly, and erector spinae posteriorly surrounding the transverse process [4]. The needle is inserted in-plane from posterior to anterior, penetrating the QL muscle to reach the fascial plane between QL and psoas major [11]. A high volume (30-40 mL) is used to achieve spread toward the thoracic paravertebral space [9]. QLB-3 is ideal for colorectal surgeries, total hip arthroplasty, and pelvic procedures. This approach targets the lumbar nerve plexus, allowing for extensive anesthetic spread to somatic and visceral fibers [15,16]. It is particularly effective for major abdominal and pelvic surgeries, such as colorectal surgeries and hip arthroplasties, as it covers the lower abdomen, pelvis, and parts of the lower extremities [17]. This method requires significantly advanced technical proficiency due to its proximity to vital structures. Potential complications of QLB-3 include lumbar plexus spread, which can lead to transient motor blockade.

QLB-4 (anterior/intramuscular QLB) involves direct intramuscular injection into QL, typically with the patient supine or in lateral decubitus [18]. Using the same US view as QLB-1, the needle is directed in-plane into the middle of the QL muscle belly [4]. Local anesthetic (around 20 mL) is injected, with spread visualized within the muscle [19]. This technique may provide somatic relief but has more variable visceral analgesia and is used less frequently for extensive abdominal procedures due to its limited spread. This block specifically targets thoracolumbar nerves before they enter the transversus abdominis plane, providing extensive somatic and visceral analgesia [1,13]. QLB-4 proves highly effective for laparoscopic and gynecological surgeries, offering broad coverage. It is primarily utilized in procedures such as hysterectomies and bowel resections that demand significant somatic and visceral analgesia. However, QLB-4 is technically demanding due to its deep anatomical location. Potential complications from QLB-4 may include inadvertent intraperitoneal injection and vascular injury [15].

Comparison of quadratus lumborum block to other abdominal blocks

To establish its efficacy and safety, QLB should be compared with other widely used anesthetic techniques, such as epidural anesthesia, the TAP block, and other regional methods. Unlike traditional approaches that target spinal nerve roots or TAP blocks, which primarily control somatic pain in the anterior abdominal wall, QLB provides both somatic and visceral analgesia. However, variations in QLB techniques necessitate a detailed comparison to inform clinical decision-making. Table 2 compares QLB with other abdominal regional anesthesia techniques, highlighting differences in analgesic coverage, type of analgesia, mechanism of action, clinical applications, advantages, limitations, and associated risks.

**Table 2:** : Quadratus Lumborum Block (QLB) vs. Other Abdominal Blocks: A Clinical Comparison

Block Type	Analgesia Coverage	Mechanism of Action	Common Clinical Applications	Advantages	Limitations	Specific Risks	References
Quadratus Lumborum Block (QLB)	Somatic and visceral analgesia (T7-L2)	Local anesthetic spread via thoracolumbar fascia to affect somatic and visceral nerves	Abdominal surgeries, cesarean sections, hip arthroplasty	Longer-lasting analgesia, covers both somatic and visceral pain, lower opioid use	Technically demanding, risk of variable anesthetic spread	Local anesthetic systemic toxicity (LAST), vascular injury, unpredictable spread	[1,11,19]
Epidural anesthesia	Somatic and visceral analgesia (Variable)	Direct neural blockade at the spinal cord level	Major abdominal, thoracic, and lower limb surgeries	Effective for major surgeries, allows continuous analgesia with catheter placement	Higher complication risk: hypotension, urinary retention, motor block	Hypotension, urinary retention, risk of dural puncture and motor block	[22-24]
Paravertebral block (PVB)	Somatic and visceral analgesia (Thoracic and lumbar)	Injection near the paravertebral space to block segmental nerves	Thoracic and abdominal surgeries, breast surgery	Targeted nerve blockade, effective for unilateral pain control	Technically challenging, risk of pneumothorax with thoracic injections	Pneumothorax, nerve damage, intravascular injection	[24,25]
Erector spinae plane (ESP) Block	Somatic and mild visceral analgesia (Variable)	Local anesthetic spread through the erector spinae muscle plane	Thoracic, abdominal, and lumbar spine surgeries	Simple and safe under ultrasound guidance, broad dermatomal coverage	Less effective for visceral pain, inconsistent spread	Inconsistent analgesic spread, less effective for deep visceral pain	[25]
Transversus abdominis plane (TAP) block	Somatic analgesia (T10-T12)	Local anesthetic spread in the TAP plane targeting anterior abdominal wall nerves	Hernia repair, appendectomy, minor laparoscopic procedures	Easy to perform, good for anterior abdominal wall pain	Limited to somatic analgesia, shorter duration compared to QLB	Limited visceral analgesia, requires large volume for effective spread	[25,26]
Transversalis fascia plane (TFPB) block	Somatic and visceral analgesia (variable, mostly lower abdomen)	Injection within the transversalis fascia plane targeting thoracolumbar nerves	Pediatric hip surgeries, abdominal procedures	Long-lasting effect, effective for lower abdominal pain control	Limited data in clinical practice, variability in effectiveness	Risk of femoral nerve blockade, difficulty in ultrasound visualization	[27-29]

Epidural anesthesia blocks neural transmission at the spinal cord, giving adequate analgesia, but it carries risks such as hypotension, dural puncture, and motor block [20]. On the other hand, QLB targets thoracolumbar nerves via the posterior abdominal wall and is less invasive. After treatment, it uses less opioid than epidurals anesthesia [21]. While QLB provides effective pain relief, its shorter duration without catheter placement makes epidural anesthesia preferable for procedures that have a prolonged duration [22].

The TAP block alleviates somatic pain by targeting the nerves in the anterior abdominal wall [23]. While TAP blocks are simpler and suitable for minor procedures, QLB provides more extensive and longer-lasting analgesia, making this technique more appropriate for surgeries involving significant visceral pain, such as bowel resections or cesarean sections [23]. Similarly, although both QLB and paravertebral blocks target the thoracolumbar nerves, QLB facilitates anesthetic spread through fascial planes, offering broader coverage. Compared to the erector spinae plane (ESP) block, QLB has deeper diffusion, resulting in greater effectiveness for visceral pain. Additionally, QLB is easier to perform, particularly under ultrasound guidance, compared to the ESP block [24].

The transversalis fascia plane block (TFPB) provides a longer-lasting analgesic effect than QLB-3 in specific settings, such as pediatric hip surgeries. TFPB also offers better ultrasonographic visualization, a higher success rate in femoral nerve blockade, and delayed first requests for analgesia. At the same time, QLB-3 remains less effective in blocking the femoral nerve due to its posterior anatomical target [25-27].

Multiple studies have compared TAP blocks and QLB across surgeries, including cesarean sections, laparoscopic procedures, hip surgeries, and abdominal hysterectomies, consistently demonstrating QLB's superior analgesia and reduced opioid requirements [28-31]. Additionally, research since 2015 has highlighted QLB's advantages over TAP blocks, including broader dermatomal coverage (T7-T12 vs. T10-T12 in ultrasound-guided TAPB), longer-lasting analgesic effects, and lower complication rates [1,3,13,32]. These benefits can be attributed to two primary mechanisms: the spread of local anesthetic to the sympathetic nerve network in the thoracolumbar plane and its diffusion into the paravertebral space, which enhances blockade duration and visceral pain control [1,13,29]. Furthermore, QLB has been associated with lower local anesthetic blood levels and prolonged analgesic effects compared to TAPB. Murouchi et al. investigated the relationship between local anesthetic blood levels and the effectiveness of QLB-2 versus TAPB, revealing that while TAPB resulted in higher anesthetic blood levels, QLB-2 provided superior pain relief [33].

Quadratus lumborum block in ERAS

An essential element of postoperative care for surgery patients involves optimal pain control, reduced opioid consumption, and early mobilization. Indeed, the ERAS protocol, which includes multimodal analgesia and early mobilization, has been shown to reduce hospital stay length and the incidence of complications following surgeries. While epidural anesthesia and nerve blocks are popular methods of pain control, they can also delay ambulation, which may slow down postoperative recovery [34]. Therefore, there is a need for less invasive regional anesthetic techniques such as interfacial plane blocks. Unlike peripheral regional blocks, which have specific neural endpoints, interfacial nerve blocks like the QLB are injected in tissue planes and target variable nerve endings depending on the spread of the local anesthetic. Over the past decade, the QLB has provided effective analgesia after several surgical procedures. Patients receiving QLB reported lower consumption of postoperative opioids and lower postoperative pain scores measured by Face, Legs, Activity, Cry, and Consolability (FLACC), Numerical Rating Scale (NRS), or Visual Analog Score (VAS), along with shorter post-anesthesia care unit (PACU) stays and longer times before the first analgesic request. Moreover, there was a lower incidence of nausea in these patients; however, no difference in vomiting or itching was reported [25-27].

The benefits of QLB for pain relief after cesarean sections in patients who have not received intrathecal morphine are notable [35,36]. A recent study by Salama et al. compared the QLB to intrathecal morphine (ITM) for postoperative pain management after cesarean sections, demonstrating that pain scores were lower at 12 and 24 hours with the QLB [37]. There was also a reduction in 48-hour morphine consumption and time to the first morphine dose. Moreover, NRS pain scores were significantly lower at 12 and 24 hours, and the time to the first analgesic dose was notably extended [35-37]. However, in patients who received intrathecal morphine for postoperative pain relief after a cesarean section, the addition of QLB did not reduce morphine consumption for PCA demands at any time point up to 24 hours. Pain scores at rest and during movement were reduced six hours after the QLB but not at any other time point up to 48 hours [38].

Meta-analyses and individual studies have reinforced the role of QLB in ERAS protocols. Jin et al. and Korgvee et al. demonstrated reduced opioid consumption and improved pain control in pooled analyses of QLB use in cesarean sections, renal, abdominal, and hip surgeries [28,39]. Despite its broad applicability, variability in outcomes remains a concern. For instance, Haskins et al. reported a lack of beneficial effects with anterior QLB in hip arthroscopic surgeries [40]. Meanwhile, Kukreja et al. found a statistically significant reduction in pain scores and opioid consumption with anterior QLB in patients undergoing hip arthroplasties [41].

In specific procedures, such as hip surgeries, systematic reviews and meta-analyses have confirmed the efficacy of QLB in reducing pain and opioid consumption over 24 hours [26,28,39,42]. Similarly, a study investigated the effectiveness of QLB for procedural and post-procedural pain management during percutaneous renal cryoablation (PRC) for renal cell carcinoma (RCC). The study noted that QLB provided effective pain control, as no patients required additional analgesics during or after the procedure, significantly decreasing the hospital stay to only four hours. This suggests that QLB could help make PRC for RCC a potential outpatient procedure [43]. A study by Baidya et al. has also indicated that QLB has been used successfully in pediatric patients undergoing pyeloplasty and laparoscopic appendectomy [44]. Murouchi utilized a bilateral QL intramuscular block on pediatric patients who underwent laparoscopic appendectomy and recorded that it was associated with adequate postoperative analgesia [33].

Safety, potential risks, and disadvantages

QLB has a favorable safety profile when performed by experienced practitioners. First, ultrasound enhances safety by providing real-time visualization of anatomical landmarks, ensuring precise needle placement and minimizing the risk of complications. When strict adherence to sterile techniques and appropriate anesthetic dosing is maintained, the risk of adverse events is significantly reduced.

However, when performing QLB injection, site infections may occur, highlighting the importance of aseptic techniques, including thorough skin preparation and sterile equipment usage [45]. Hematoma formation in the quadratus lumborum region is another complication, particularly in patients with coagulopathies or those on anticoagulants [46]. A rare but serious risk is LAST, which can arise from inadvertent intravascular injection of anesthetic [4]. Although the slow absorption of anesthetic in the fascial plane lowers the likelihood of LAST in QLB, strict adherence to recommended dosing limits and careful patient monitoring remain essential [47].

Another challenge with QLB is the potential for uncontrolled anesthetic spread. Due to the anatomical proximity of the thoracolumbar fascia at the lateral border of the quadratus lumborum muscle and its closeness to the transversalis fascia, there is a risk of anesthetic diffusion into the lower thoracic paravertebral space, the TAP, or anteriorly into the psoas major muscle, which could potentially affect the lumbar plexus [12]. While QLB-1 is effective for somatic wall pain, it is less effective for visceral pain in abdominal surgeries, often necessitating supplemental systemic opioids or alternative analgesic approaches [12].

Due to the steep needle trajectory, QLB-3 poses unique risks, including the increased likelihood of lumbar artery vascular injuries [12]. Additionally, placing patients in the lateral decubitus position for the procedure can be potentially hazardous, particularly in hemodynamically unstable patients. An unintended femoral nerve block is another possible complication of QLB-3, which could impair lower limb mobility and delay postoperative ambulation [12].

QLB-4, which targets the lumbar plexus, poses a risk of inadvertent plexus blockade without clearly identifying nerve structures, making it a less commonly used approach [12]. Furthermore, retroperitoneal hematoma formation has been reported, adding another layer of risk [48].

Beyond anatomical risks, variability in QLB effectiveness has been observed across different approaches and patient populations. Studies have shown that anterior, posterior, and lateral QLB techniques yield differing outcomes, highlighting the need to carefully select the appropriate approach to optimize efficacy. Additionally, tissue compliance influences local anesthetic spread, making standardization of QLB techniques challenging [26-30].

Other studies highlighted that while QLB requires meticulous ultrasound guidance to avoid major blood vessels and nerves, the TFPB offers superior ultrasonographic visualization, making it easier to achieve consistent outcomes than QLB [29,30]. The technical difficulty of QLB remains a limitation since precision is critical for preventing complications and ensuring adequate analgesia. 

Despite its advantages in reducing opioid use, the efficacy of QLB in specific scenarios remains uncertain. Studies indicate that the variability in local anesthetic spread depends more on tissue compliance than on needle placement, further complicating efforts to standardize QLB techniques [26,27]. These factors underscore the need for continued research to refine QLB applications, enhance safety, and improve reliability in clinical practice.

Quadro-iliac plane block and future directions of the quadratus lumborum block

The emerging anesthesia technique, quadro-iliac plane block (QIPB), was developed to provide analgesia to the lumbosacral, hip, and abdominal regions. With this block, local anesthetic is administered to the posterior aspect of the quadratus lumborum muscle at its origin from the iliac crest.

A cadaveric study by Tulgar et al. investigated the QIPB procedure using the spread of dye in the deep interfascial plane of the erector spinae muscle, with extensive staining that was observed on the anterior and posterior aspects of the QL muscle [49]. The dye traversed along the transversalis fascia, infiltrating the retroperitoneal fat tissue, staining the ilioinguinal and iliohypogastric nerves bilaterally. With this technique, preliminary evidence indicates more extensive spread of anesthetic agents to target areas and staining of the lumbar plexus on both sides, indicating that the QIPB method has the potential to provide comprehensive analgesia to targeted regions [49].

A case report performed by Turan et al. with a 43-year-old female patient shows a unilateral QIPB performed using 50 mg of 0.25% bupivacaine under ultrasound guidance. With this technique postoperative discomfort had decreased from an NRS score of 8 to a 3 at the 6 and 12 hour marks when the patient was at rest, indicating QIPB’s effectiveness at managing postoperative pain in high-risk patients with opioid use risk [50].

Despite the increasing use of QLB in perioperative pain management, several aspects require further research to optimize its clinical application and establish it as a standard of care in regional anesthesia. One crucial area of investigation is the optimization of injection volumes and anesthetic spread. While studies suggest that QLB achieves both somatic and visceral analgesia through diffusion along the thoracolumbar fascia, variability in spread remains a concern. Additional research should focus on high-resolution imaging studies to accurately map the distribution of the anesthetic and determine the ideal volume-to-coverage ratio, ensuring both efficacy and safety while minimizing LAST.

Another key area is integrating QLB into ERAS protocols. Current evidence suggests that QLB reduces opioid consumption, improves postoperative mobility, and shortens hospital stays, particularly in abdominal and colorectal surgeries. However, additional randomized controlled trials (RCTs) comparing QLB with TAP blocks and epidural anesthesia are necessary to define its role in multimodal analgesia strategies and confirm its benefits in ERAS settings.

The role of QLB in obstetric anesthesia, especially for cesarean sections, also requires further investigation. While initial studies show effective postoperative analgesia, the comparison between QLB and intrathecal morphine remains a debated topic. Future trials should assess opioid-sparing effects, maternal recovery times, and neonatal safety outcomes to determine whether QLB can replace intrathecal opioids as a primary technique for cesarean deliveries.

Additionally, research into pediatric applications of QLB is essential. Although QLB has shown promising results in pyeloplasty, laparoscopic appendectomy, and hernia repair, data on optimal dosing, duration of action, and long-term safety in pediatric patients remain limited. Comparative studies between QLB and traditional nerve blocks, such as the ilioinguinal-iliohypogastric block, could further clarify its advantages and establish standardized protocols for pediatrics.

By addressing these research gaps, QLB has the potential to become the preferred regional anesthesia technique for abdominal and pelvic surgeries. The standardization of injection techniques, expansion of clinical trials, and long-term safety evaluations will be critical in elevating QLB to the status of a first-line anesthetic technique in modern perioperative care.

## Conclusions

The QLB has emerged as a highly effective regional anesthesia technique, offering both somatic and visceral analgesia with superior pain control compared to traditional abdominal blocks. As regional anesthesia becomes increasingly integrated into ERAS protocols, QLB stands out for its ability to reduce opioid consumption, improve recovery times, and enhance patient outcomes. With the widespread availability of ultrasound technology, performing QLB is now more accessible, making it a viable alternative to epidural anesthesia and TAP blocks. Given its clinical benefits, QLB should be regarded as the new gold standard for abdominal regional anesthesia. To facilitate its adoption, anesthesia residency programs must prioritize the teaching of abdominal blocks, particularly QLB, ensuring that the next generation of anesthesiologists is proficient in this technique. Expanding QLB training and research will be critical in shaping the future of perioperative pain management.
